# Effects of ***β***-Adrenoceptor Subtypes on Cardiac Function in Myocardial Infarction Rats Exposed to Fine Particulate Matter (PM_**2.5**_)

**DOI:** 10.1155/2014/308295

**Published:** 2014-08-12

**Authors:** Yuping Gao, Jiyuan Lv, Yuanyuan Lin, Xuewen Li, Lixia Wang, Yanping Yin, Yan Liu

**Affiliations:** ^1^First Hospital of Shanxi Medical University, Taiyuan 030001, China; ^2^The Affiliated Dayi Hospital of Shanxi Medical University, Taiyuan 030032, China

## Abstract

The pathophysiological mechanisms of heart failure (HF) stems were mainly from longstanding overactivation of the sympathetic nervous system and renin-angiotensin-aldosterone system. Recent studies highlighted the potential benefits of *β*1-adrenoceptor (*β*1-AR) blocker combined with *β*2-adrenergic receptor (*β*2-AR) agonist in patients with HF. Long-term exposure to fine particulate air pollution, such as particulate matter ≤ 2.5 *μ*m in diameter (PM_2.5_), has been found associated with acute myocardial infarction (AMI) which is the most common cause of congestive HF. In this study, we have investigated the effect of combined metoprolol and terbutaline on cardiac function in a rat model of AMI exposed to PM_2.5_. Our results demonstrated that short-term exposure to PM_2.5_ contributes to aggravate cardiac function in rats with myocardial infarction. The combined use of *β*1-AR blocker and *β*2-AR agonist is superior to *β*1-AR blocker alone for the treatment of AMI rats exposed to PM_2.5_. The combination of *β*1-AR blocker and *β*2-AR agonist may decrease the mortality of patients with myocardial infarction who have been exposed to PM_2.5_.

## 1. Introduction

*β*-Adrenergic receptor (*β*-adrenoceptor, *β*-AR) is an important member of sympathetic nervous system, which plays an important role in regulating the heart function by mediating the physiological effect of catecholamines [1]. *β*-AR has at least 3 subtypes (*β*1, *β*2, and *β*3), and the former two are important in the regulation of excitation-contraction coupling of myocardium and have positive inotropic response related to cell growth  [2]. Activation of *β*1-adrenergic receptor (*β*1-AR) can induce myocardial cell hypertrophy, apoptosis, cell necrosis, and myocardial remodeling activity in the earlier stage of heart failure. *β*2-Adrenergic receptor (*β*2-AR) signalling can also grant protection against programmed cell death in myocytes, countering the proapoptotic action of *β*1-adrenoceptor stimulation via a Gi-mediated process [3–5]. In contrast, *β*3-adrenergic receptor (*β*3-AR) is very rare in human cardiac myocytes, which may appear only in severe heart failure. Combined use of *β*1-AR blocker and *β*2-AR agonist has been proposed to treat chronic heart failure [6], and they have demonstrated improved cardiac function in rats with heart failure, ventricular remodeling, and myocardial apoptosis [7, 8].

Recently, air pollution has been found to increase risks for a wide range of diseases. Studies indicate that in recent years exposure levels have increased significantly particularly in rapidly industrializing countries with large populations. Exposure to particulate matter with diameter less than or equal to 2.5 *μ*m (PM_2.5_) has been implicated as a risk factor on respiratory system and cardiovascular system [9, 10]. Particularly, it has been observed that long-term [11–13] or short-term [14, 15] PM exposures might trigger acute ischemic heart disease. The possible mechanisms include activation of the sympathetic nervous system, oxidative stress, inflammation, atherosclerosis, and destruction of blood coagulation, thus affecting the cardiovascular system, for instance, ischemic stroke [16, 17], echocardiography (ECG) ST-segment depression [18, 19], increased plasma viscosity [20], increased circulating markers of inflammation [21, 22], and changes in heart rate variability [23, 24].

Until now, to the best of our knowledge, there were no studies on the effect of *β*-AR subtypes on cardiac function in rats with myocardial infarction after administration of PM_2.5_. The aim of this study is to investigate the effect of metoprolol (a *β*1-AR blocker) and terbutaline (a *β*2-AR agonist) on cardiac function in a rat model of myocardial infarction rats after exposure to PM_2.5_.

## 2. Materials and Methods

### 2.1. Animals

Adult male Sprague-Dawley rats weighing 200–250 g were obtained from the Laboratory Animal Center of Shanxi Medical University, China. All rats were housed in light-controlled animal quarters with temperature of 23 ± 2°C and were provided free access to food and libitum. This study was approved by the Bioethical Committee of Shanxi Medical University and all the procedures followed the National Guideline for the Care and Use of Laboratory Animals.

### 2.2. Acute Myocardial Infarction and Study Groups

The rats were anesthetized with sodium pentobarbital (60 mg/kg, i.p.) and underwent sterile surgery. Acute myocardial infarction (AMI) was induced by ligating the left anterior descending (LAD) coronary artery for the study group but without ligating the vessel for sham surgery group as previously described [25, 26]. In order to investigate the effect of PM_2.5_, rats were randomly assigned to two different study arms: one study arm without PM exposure and the second study arm with PM exposure.

In the first study, rats were divided into five groups (*n* = 6 in each group): sham, AMI, metoprolol (AstraZeneca Pharmaceutical Co., Ltd., Wuxi, China, 20 mg/kg body weight twice a day), terbutaline (AstraZeneca Pharmaceutical Co., Ltd., Wuxi, China, 2 mg/kg body weight twice a day), and combined use of metoprolol (20 mg/kg body weight twice a day) and terbutaline (2 mg/kg body weight twice a day). Metoprolol and terbutaline were administered orally by gavage.

For the second study, rats were exposed to PM_2.5_ (Label # 2783, National Institute for Standards and Technology, USA). All the rats were anesthetized with sodium pentobarbital (60 mg/kg, i.p.) and placed supine with extended neck on an angled board [27]. PM_2.5_ at a final concentration of 40 mg/kg body weight was administrated through a cannula inserted into the trachea just above the bifurcation [28]. This intratracheal instillation procedures were done within 3 seconds. Then, rats were divided into five groups (*n* = 10 in each group): sham, AMI, metoprolol (20 mg/kg body weight twice a day), terbutaline (2 mg/kg body weight twice a day), and combined use of metoprolol (20 mg/kg body weight twice a day) and terbutaline (2 mg/kg body weight twice a day).

### 2.3. *In Vivo* Measurement of Cardiac Function

Rats were anesthetized with sodium pentobarbital (60 mg/kg, i.p.), a polyethylene catheter connected with a pressure transducer was inserted into the left ventricular cavity via the right carotid artery [25]. Left ventricular systolic pressure (LVSP) and left ventricular diastolic pressure (LVDP) were digitally processed via hemodynamic analyzing system (Powerlab Hardware, AD Instruments) [29]. Maximal positive and negative values of the instantaneous first derivative of LVP (+dP/dt_max⁡_ and −dP/dt_max⁡_) were derived by computer algorithms.

### 2.4. ECG and Hemodynamic Measurements

To evaluate the impact of metoprolol and terbutaline on cardiac performance, ECG was performed on all rats before and after MI, as well as after administration of each medication. ECG was performed under anesthetizing with sodium pentobarbital (60 mg/kg, i.p.) to assess left ventricular function as previously described [30]. Images were obtained with Sonos 5500 (Philips Medical Systems, Washington, USA) fitted with probe (frequency of 8 MHz), which generated two-dimensional images at a rate of 100/s, and then analyzed short and long axis images of left ventricle. End-diastolic dimension (EDD) and end-systolic dimension (ESD) were measured. Ejection fraction (EF) was obtained by M-mode echocardiography. Fractional shortening (FS) was calculated by the following equation: FS = [(EDD − ESD)/EDD × 100].

### 2.5. Western Blotting for Expression of Bcl-2 and Bax Protein

Protein was extracted from frozen left ventricular tissue. Bovine serum albumin as a standard protein concentration was determined by the bicinchoninic acid protein assay (Pierce Rockford, IL). Equal proteins were loaded on 12% SDS-PAGE which were confirmed by Coomassie blue staining. The sample volume is 40 *μ*g. Proteins were transferred to a polyvinylidene membrane [31, 32]. Rat anti-Bcl-2 monoclonal antibody (Santa Cruz Biotechnology; Santa Cruz, CA) and mouse anti-Bax monoclonal antibody (Santa Cruz Biotechnology; Santa Cruz, CA) were used for Bcl-2 and Bax detection, respectively. The bands were visualized by Phototope-HRP Western blot detection kit (New England Biolabs, Beverly, MA) and were scanned by GS-700 densitometer (Bio-Rad Company, Hercules, CA) and then quantified by quantitative procedure. Optical density of the tissue samples was normalized to the control sample in arbitrary densitometry unit.

### 2.6. Determination of Myocardial Apoptosis by TUNEL

To determine myocardial apoptosis, cardiac tissues from the MI group, sham group, MI + meto group, MI + terbu group, and MI + meto + terbu group were perfused first with 0.9% NaCl for 5 min and then with 4% paraformaldehyde in PBS (pH = 7.4) for 20 min. Ischemic regions were cut to 5 mm thickness at a level of left ventricular long axis and further fixed in 4% paraformaldehyde in PBS for 24 h at room temperature. Fixed tissues were embedded in a paraffin block and two slides at 5 *μ*m thickness were cut from each tissue block [33]. The slides were processed for a TUNEL assay. An In Situ Cell Death Detection Kit (Boehringer Mannheim, USA) was used according to the manufacturer's instructions. Briefly, the slides were treated with H_2_O_2_ and incubated with the reaction mixture containing TdT (terminal deoxyribonucleotide transferase) and FITC-conjugated dUTP at 4°C overnight. One part of labeled DNA was visualized with fluorescent microscopy. Another part was incubated subsequently with alkaline phosphatase (AP) conjugated anti-FITC antibody for 1 h at 37°C and then visualized using AP-Red as the chromogen. Finally, the labeled DNA was detected with medical image analysis system. For negative control, TdT was omitted from the reaction mixture. Assays were performed in a blinded manner.

### 2.7. Myocardial Glutathione (GSH) Measurement

Increased oxidative stress in the myocardium has been observed in heart failure animals; the GSH/GSSG ratio, a measure of overall cellular oxidative stress, was calculated to evaluate the degree of heart failure. Fresh left ventricular myocardial tissue was homogenized. The supernatant was collected for measuring total GSH using a GSH reductase-coupled enzymatic assay on a SPR-960B microplate reader (Changchun Sainuomaide Medical Technology Co., Ltd.). Total GSH was calculated from a standard curve of purified GSH, and glutathione disulfide (GSSG) was measured by masking the reduced GSH with 2-vinyl pyridine in the enzymatic assay. Total GSH = 2 × GSSG + GSH, GSH/GSSG was calculated [34].

### 2.8. Statistical Analyses

SPSS 17.0 statistical software package was used for the statistical analysis. All quantitative data were presented as mean ± SD and analyzed by using the least-significant difference test of analysis of variance. Differences were considered statistically significant at *P* < 0.05. Survival rates in each group were analyzed by the Kaplan-Meier method [35].

## 3. Results

### 3.1. Cardiac Performance

**Figure 1:**
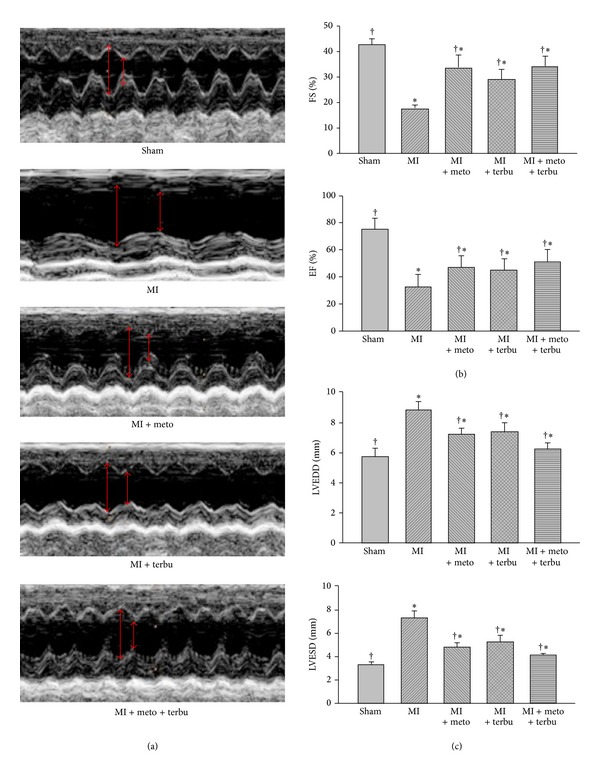
Echocardiographic imaging demonstrated improved myocardial function after treatment of metoprolol, terbutaline, or combined metoprolol and terbutaline. (a) Representative M-mode short axis views in sham, MI, and MI groups after treatment of metoprolol, terbutaline, or combined metoprolol and terbutaline. (b) Percentage of fractional shortening (FS%) and ejection fraction (EF%) calculated from short axis views. (c) Values of LVESD and LVEDD in sham, MI, and MI rat after treatment of metoprolol, terbutaline, or combined metoprolol and terbutaline. *P < 0.05 versus sham. ^†^ *P* < 0.05 versus MI.

Representative M-mode recordings of diastolic and systolic left ventricular (LV) diameters were shown in Figure 1. Measurements taken from these views revealed LAD ligation resulted in cardiac chambers dilatation, wall thinning, and descending myocardial function represented by fractional shortening (%FS) and ejection fraction (%EF). Compared with the sham control, the LV fractional shortening and ejection fraction of MI rats were significantly improved after treatments of metoprolol, terbutaline, or combined metoprolol and terbutaline. In particular, the effect of combined metoprolol and terbutaline is better than that of metoprolol or terbutaline alone.

### 3.2. Hemodynamic Parameters

**Figure 2:**
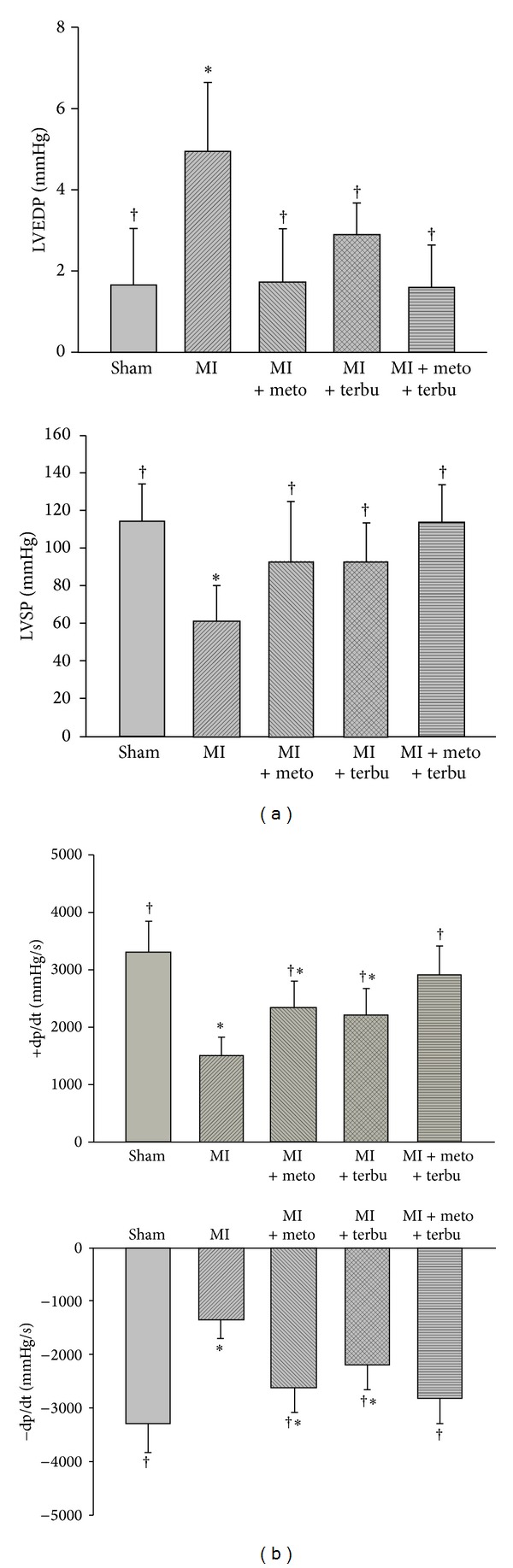
Improved myocardial function after treatment of metoprolol, terbutaline, or combined metoprolol and terbutaline. (a) Left ventricular end-diastolic pressure (LVEDP) and left ventricular peak systolic pressure (LVSP) in sham, MI, and MI groups after treatment of metoprolol, terbutaline, or combined metoprolol and terbutaline. (b) Representative recordings of positive and negative values of the instantaneous first derivative of left ventricular pressure (±dP/dt) in sham, MI, and MI rats that underwent gavage of metoprolol, terbutaline, or metoprolol plus terbutaline. *P* < 0.05 versus sham. ^†^ *P* < 0.05 versus MI.

The hemodynamic parameters of rats are presented in Table 1. LAD ligation resulted in hemodynamic changes indicative of LV dysfunction (Figure 2). Compared with sham group, MI rat exhibited a marked decrease in left ventricular peak systolic pressure (LVSP), LV end-diastolic pressure (LVEDP), as well as impairment in LV contraction and relaxation (±dP/dt). LVSP, LVEDP, and ±dP/dt were increased after treatments of metoprolol, terbutaline, or combined metoprolol and terbutaline. Myocardial function was significantly improved especially after combined use of metoprolol and terbutaline, compared to that of metoprolol or terbutaline alone.

### 3.3. Protein Expressions

**Figure 3:**
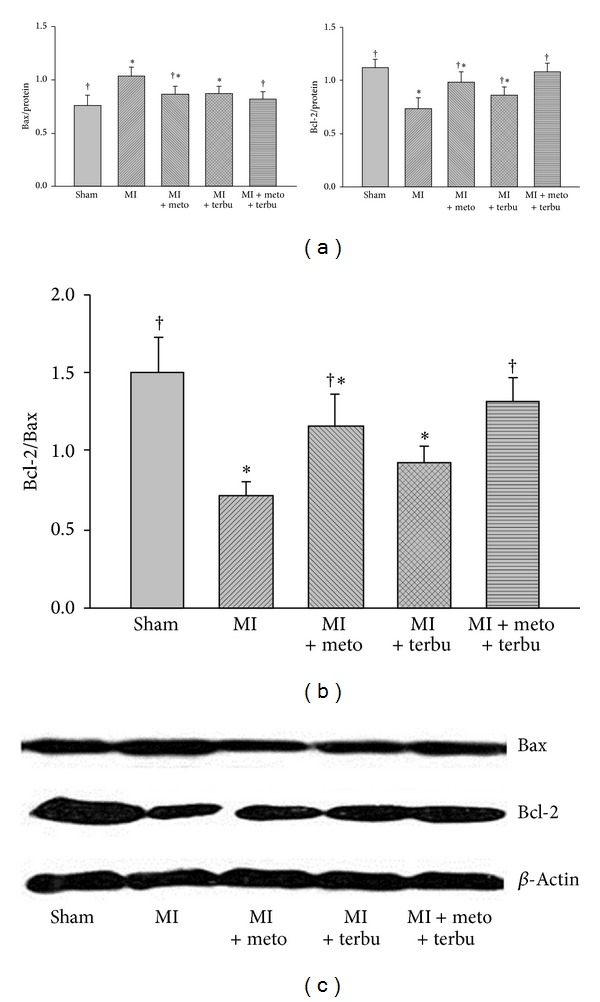
Effects of different medications on myocardial Bax, Bcl-2, and Bcl-2/Bax in MI rats. (a) Expression of Bcl-2 and Bax in sham and MI rats treated with metoprolol, terbutaline, or combined metoprolol and terbutaline. (b) Expression of Bcl-2/Bax in sham and MI rats treated with metoprolol, terbutaline, or combined metoprolol and terbutaline. *P < 0.05 versus sham. ^†^ *P* < 0.05 versus MI. (c) Expressions of Bax and Bcl-2 in different treatment groups.

The proapoptotic protein (Bax) and antiapoptotic protein (Bcl-2) were determined by western blot (Figure 3). Compared with the sham group, Bcl-2 protein expression was decreased in the AMI rats. This was associated with an increase in Bax protein. So, Bax to Bcl-2 ratio (Bcl-2/Bax) was decreased in AMI rats. Treatments of metoprolol and terbutaline prevented the decrease of Bcl-2 protein and increase of Bax and decrease of Bcl-2/Bax in AMI rats. The therapeutic effect of combined metoprolol and terbutaline is significantly better than metoprolol or terbutaline alone.

### 3.4. Myocardial Apoptosis

**Figure 4:**
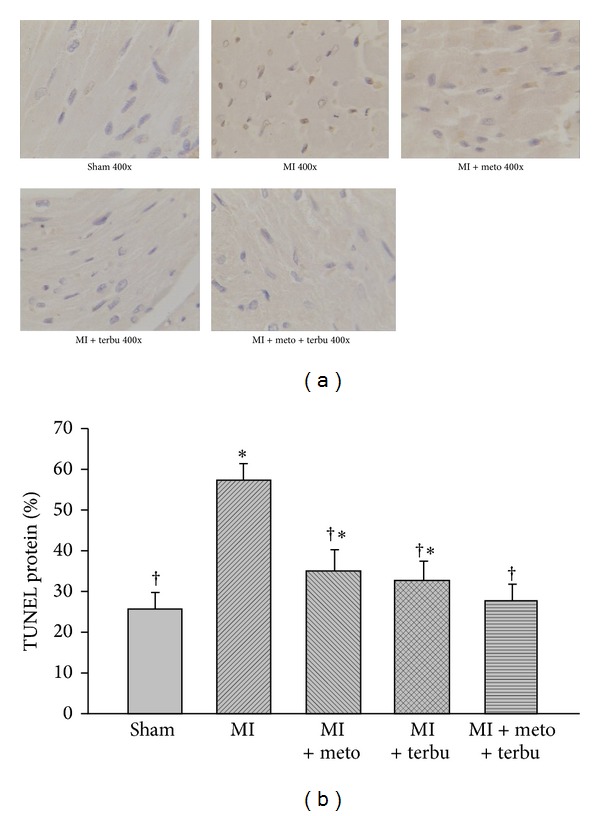
Effects of drug interventions on myocardial apoptosis in MI-induced CHF rats. Myocardial apoptosis was detected by TUNEL staining. (a) Representative photomicrographs of TUNEL staining are shown in sham group, MI group, and MI-induced CHF rats treated with metoprolol, terbutaline, and metoprolol plus terbutaline. (b) Quantification of TUNEL positive cardiomyocytes. *P < 0.05 versus sham. ^†^ *P* < 0.05 versus MI.

Myocardial apoptosis in the MI group was significantly higher than that of the sham group. However, the degree of apoptosis after drug interventions was significantly lower in the MI + meto, MI + terbu, and MI + meto + terbu groups than in the MI group (Figure 4). The changes were most marked in the combined metoprolol and terbutaline-treated AMI rats.

### 3.5. Myocardial GSH

**Figure 5:**
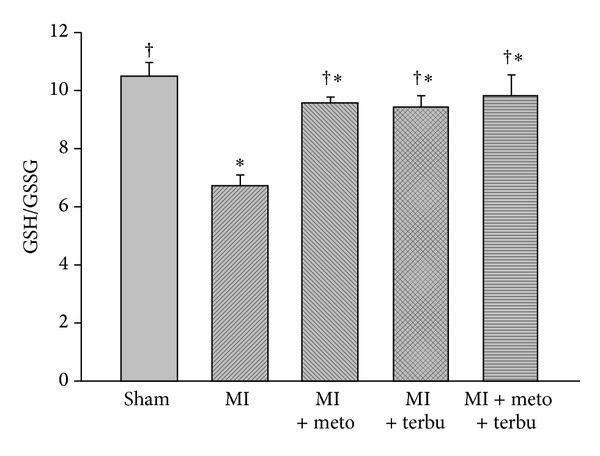
Effects of MI-induced CHF and drug interventions on left ventricular GSH/GSSG. The statistical bar graphing showing that left ventricular GSH/GSSG from sham and CHF rats treated with metoprolol, terbutaline, and metoprolol plus terbutaline. *P < 0.05 versus sham. ^†^ *P* < 0.05 versus MI.

The myocardial tissue GSH/GSSG was determined in sham and AMI animals (Figure 5). Compared with the sham animals, left ventricular GSH/GSSG was decreased in AMI rats. Drug interventions attenuated the decrease of GSH/GSSG. The changes were most marked in the combined metoprolol and terbutaline-treated AMI animals.

### 3.6. Survival Analysis

**Figure 6:**
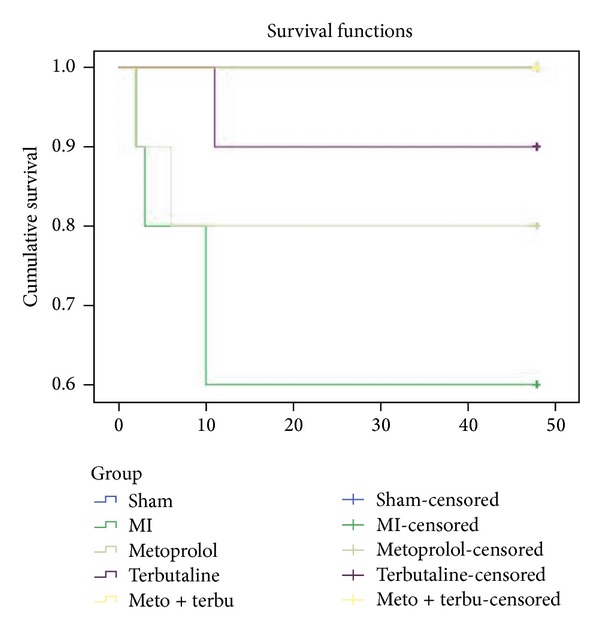
Kaplan-Meier survival curve comparing sham with CHF rats which were treated with metoprolol, terbutaline, and metoprolol plus terbutaline. The graph showed that the survival rates were significantly lower in CHF group than the other treated groups.

Rats exposure to PM_2.5_ was found with significant ventricular arrhythmia along with a further decrease of heart rate. Forty-eight hours after medication intervention, survival rates were 100%, 60%, 80%, 90%, and 100%, respectively. No significant differences were found between sham group and medication administrated groups (Table 1). There were significant differences between MI nontreatment group and the other treated groups. The Kaplan-Meier survival risk analysis was presented in Figure 6.

## 4. Discussion

In the present study, significant increase of the ±dp/dt and LVSP and decrease of LVEDP were found in the single or combined use of metoprolol and terbutaline, compared with those in the untreated AMI group. In addition, combined use of metoprolol and terbutaline improved cardiac function significantly more than that of the treatment with metoprolol alone. In addition, cardiac remodeling was evidenced by the reduced FS and EF, as well as progressive dilation of the left ventricle in the AMI rats. This was associated with the increase of LVEDD and LVESD. Metoprolol or terbutaline alone or combined use of metoprolol and terbutaline can significantly ameliorate cardiac remodeling by reducing LVEDD and LVESD and increasing the FS and EF of left ventricle. The combined use of metoprolol and terbutaline had a better therapeutic response than that of metoprolol or terbutaline alone.

Numerous previous clinical studies have confirmed that long-term treatment with *β*1-AR blocker could significantly improve cardiac function; therefore, they reduce the total mortality and morbidity in patients with heart failure [36–38]. Our present study is consistent with the previous study which demonstrated that treatment with *β*-AR blocker could improve ventricular function and reduce cardiomyocyte apoptosis in myopathic turkeys [39]. AMI or HF is associated with a decrease in the antiapoptotic Bcl-2 protein and an increase in proapoptotic Bax protein [40]. Our results further demonstrated that combined metoprolol and terbutaline prevented the changes in both Bcl-2 and Bax proteins in MI-induced heart failure. Additionally, we observed that combined metoprolol and terbutaline significantly reduced Bcl-2/Bax ratio. In the present study, by using TUNEL staining, which is the standard method for the evaluation of apoptosis, we found that metoprolol and terbutaline significantly inhibited the cell apoptosis and the combination therapy with metoprolol and terbutaline had more significant effect.

Furthermore, we found that combined use of metoprolol and terbutaline suppressed oxidative stress-mediated myocyte apoptosis by increasing GSH/GSSG, which further confirmed that *β*1-AR blocker and *β*2-AR agonist may play an important role in the antioxidant effect as well as in AMI. This is consistent with the previous rat study, which demonstrated that carvedilol could attenuate myocardial inflammation and decrease the severity of myocarditis by the antioxidant action [8].

Few studies found that *β*1-AR and *β*2-AR had significantly different signal transduction pathways [41]. *β*2-AR signal transduction pathways conclude both Gs-AC-PKA pathway and Gi-PI3K-AKT pathway. Therefore, activation of *β*2-AR to a certain extent can inhibit apoptosis and improve cardiac function [5, 42].

Additionally, our studies demonstrated that short-term exposure to PM_2.5_ contributed to aggravate cardiac function in myocardial infarction rats. The mortality rate decreased after treatment of *β*1-AR blocker and *β*2-AR agonist. Many recent studies suggested that long-term exposure to low concentration of PM_2.5_ might alter vasomotor tone and induce vascular inflammation or atherosclerosis, increased risk of ischemic disease, and death due to pulmonary and systemic oxidative stress [9, 11, 14, 43]. Short-term PM exposures may also trigger acute ischemic heart disease events [14] which may be related to inflammation and acute complications of atherosclerosis and, thus, lead to atherosclerotic plaque rupture, thrombosis, and precipitation of acute ischemic events [15].

In our study, significant reversal of cardiac remodeling and improvement of hemodynamic function were found in the rat model of AMI after treatment of *β*1-AR blocker and *β*2-AR agonist. Our results suggested that reduction of myocardial oxidative stress by *β*1-AR blocker and *β*2-AR agonist plays an important role in AMI or heart failure. Combining *β*1-AR blocker with *β*2-AR agonist is better than the use of *β*1-AR blocker or *β*2-AR agonist alone. We conclude that *β*1-AR blocker and *β*2-AR agonist might be important clinical approach in the treatment of heart failure. Particularly, *β*1-AR blocker and *β*2-AR agonist may decrease the mortality after exposure to PM_2.5_.

## Figures and Tables

**Table 1:** Pairwise comparisons of Kaplan-Meier survival risk analysis.

Group	Sham		MI		Metoprolol		Terbutaline		Meto + terbu	
	*χ* ^2^	*P*	*χ* ^2^	*P*	*χ* ^2^	*P*	*χ* ^2^	*P*	*χ* ^2^	*P*
Sham			4.739	0.029∗	2.110	0.146	1.000	0.317		
MI	4.739	0.029∗			0.736	0.391	2.592	0.107	4.739	0.029∗
Metoprolol	2.110	0.146	0.736	0.391			0.454	0.501	2.110	0.146
Terbutaline	1.000	0.317	2.592	0.107	0.454	0.501			1.000	0.317
Meto + terbu			4.739	0.029∗	2.110	0.146	1.000	0.317		

*P < 0.05 versus sham.
